# Concept of the knowledge-based city logistics: Problems and solutions

**DOI:** 10.1371/journal.pone.0305563

**Published:** 2024-06-25

**Authors:** Stanisław Iwan, Natalia Wagner, Kinga Kijewska, Sidsel Ahlmann Jensen

**Affiliations:** 1 Faculty of Economics and Transport Engineering, Maritime University of Szczecin, Szczecin, Poland; 2 Department of Technology and Innovation, The Institute of Transport Economics TØI, Oslo, Norway; Islamic University of Madinah, SAUDI ARABIA

## Abstract

Efficient city logistics is essential to build smart sustainable cities where inhabitants’ well-being is a priority. Meanwhile, despite the great importance of city logistics processes, their improvement is problematic for many cities. Although solutions from the field of emerging technologies are more and more often used, the question is whether implementing technological tools and filling cities with sensors is a sufficient solution that can solve the problems of intensely growing urban freight transport. The aim of the paper is to examine the role of knowledge management in city logistics and identify barriers to the implementation of knowledge-based city logistics. A key element of the research procedure was an expert survey, to which 31 international experts specialising in city logistics issues were invited, characterised by extensive experience working on research projects in the area of interest. Four knowledge management processes have been transferred to the city logistics area. The results of the study show that the difficulties are observed mainly in the processes of data gathering and knowledge acquisition. The main reason for difficulties in that area is the reluctance of city users, retailers, transport and logistics operators to share information. Identifying these processes as the most problematic is a valuable hint for logistics managers, municipalities and academics. To improve knowledge-based city logistics, it is therefore necessary to focus on these processes and look for the best solutions and new forms of organisational and business support. The solution to the problems identified in the study is the proposal to create a city logistics collaborative knowledge base which is a combination of an IT tool ‐ the CL knowledge management platform, and the Freight Quality Partnership.

## Introduction

Today, social and economic life is mainly concentrated in cities. In both developed and developing countries, cities are growing and attracting new residents. It is estimated that by 2050, almost 70 per cent of the world’s population will live in urban areas [1]. This will have consequences, among other things, for the organisation of transport in the city. A larger population is expected to result in faster growth in demand for freight transport and logistics services than for passenger transport [2]. Such rapid urban development requires effective city management, taking into account all aspects of the lives of its inhabitants and the functioning of economic and social actors. It is necessary to ensure good market conditions and infrastructure for transport and logistics operations, as well as to carry out in-depth studies taking into account the problems observed in city logistics.

This study combines two research fields: city logistics (CL) and knowledge management (KM). The first research field in which the study is embedded − CL − is one of the most important contemporary fields of urbanization [3]. The second is KM, which is understood as “processes of identifying, gathering and reinforcing knowledge” [4]. KM is a well-developed concept whose mechanisms and processes have been extensively studied for decades and conclusions are recommended for business practice [5]. Proper KM is considered one of the key success factors for organisations operating in today’s market [6]. Typically, recommendations on how to effectively manage knowledge are addressed to organisations, and in particular to business entities. In this study, however, this concept has been transferred to CL.

It is important to integrate the achievements of KM to a greater extent into the scientific discussion on the further development of CL. Stronger inclusion of KM approach can help cities leverage the synergies effects and create better solutions for city users. On the one hand, the study applies KM processes and rules, and on the other hand, it studies the nature of city CL projects and activities. This approach is in line with the spirit of CL considered to be a multi-disciplinary and complex field, which encompasses managerial, social and engineering aspects [7].

Numerous studies are known from the literature showing the possible applications of emerging technologies in CL and the benefits they bring. What is still lacking, however, is a broader view of the knowledge held ‐ both tacit and explicit ‐ and its flows between the different actors operating in the city. This is the task undertaken in this research.

The study focuses on the characteristics of CL, in particular on topics related to building resources of knowledge about the functioning of the city, i.e. the use of data analysis, including data mining processes related to the freight traffic in the urban areas, mapping the needs of stakeholders, and new city infrastructure decisions. It covers both management and technical issues, such as the use of methods and equipment for obtaining data from CL, the coordination of activities among city actors, and the optimisation of operational decisions taken by city stakeholders. In recent years, several studies have been carried out related to issues of data availability in CL [8, 9]. However, the problem of data availability is still not addressed to a satisfactory level for CL actors, who have a direct impact on the functioning of CL, mainly local authorities and freight transport operators. A way to solve it may be to take a broader view and treat it as part of proper KM in CL.

The aim of the paper is to examine the role of knowledge management in city logistics and identify barriers in the implementation of knowledge-based city logistics. The links between KM processes, on the one hand, and city logistics activities, on the other, were examined, and the difficulties encountered by entities involved in city logistics management were identified. This has provided a better understanding of the area under study in both a strategic and operational context. As part of the research, a questionnaire survey was sent to 31 international experts with substantial experience in city logistics projects and activities. Gathering and analyzing the opinions of a wide group of experts who have unique experience in conducting research projects in the field of CL is a great advantage of this study.

This study is a part of a broader research work on the concept of knowledge-based CL. The first partial results focused on the presentation of the findings of the literature review and research projects and the creation of a definition of knowledge-based CL [10]. Knowledge-based CL is understood as “*knowledge-based city logistics is an approach focusing on data collection and transformation to the information resources for efficient city logistics measures implementation and development and to optimize the freight flows at the urban areas*, *paying the special attention on the real-time data resources*, *information processes and knowledge sharing*” [11]. This paper is a continuation of the topic and addresses the issues of identification and analysis of difficulties encountered in CL processes and their analysis from the perspective of KM.

The remaining parts of this paper are organized as follows. The literature review section presents selected issues related to KM mechanisms and processes and the specificity of CL, which was the basis for presenting the concept of knowledge-based logistics. Methodology section characterises the research method that was used. Research results section analyses the results of an expert survey on CL processes in which KM is used and the difficulties encountered in this area. Discussion section proposes a solution to the identified problems ‐ a collaborative knowledge base in the field of CL. The paper concludes with directions for future research.

## Relationships between knowledge management and city logistics ‐ literature review

CL is a discipline specialized to cope with the sustainability problems encountered in urban freight transport [12]. Its aim is the ‘integration of existing resources in order to solve problems arising from the motorisation index increase in the city’ [13]. Definitions of CL known from the literature underline its several important dimensions, i.e. proper planning for goods distribution within a city [14], application of innovative technologies and sustainability [15], and emphasis on achieving the efficiency of logistics processes [16]. Despite the great importance of CL processes, the improvement of urban freight transport is still a challenge for many cities [17]. Topics and solutions addressed by CL include, among others, cooperation with city stakeholders, strategic decision-making (e.g. for new logistics infrastructure), traffic data acquisition and management, providing freight services for users with different requirements (e.g. citizens and tourists), the use of new business models to improve freight traffic flow, crowdsourcing, consolidation hubs, or using electric vehicles and bikes [7, 18–22]. In addition to the long-established CL issues that have been developed, such as those related to ensuring road safety [23], completely new solutions are also emerging. One of the latest trends in urban freight transport is the use of micromobility solutions [24]. There are still dynamically growing door-to-door deliveries linked to the development of business-to-consumer e-commerce [25], which must be supported by efficient management of last-mile city logistics. CL must meet even more stringent time standards for on-demand deliveries linked to quick-commerce and the new *dark store* format [26].

The technological development contributes to more and more commonly used new technology-based solutions in CL, integrating CL with the smart city concept. Such solutions include, for example, ICT (Information and Communication Technology) systems and ITS (Intelligent Transport Systems) which allow to collection ‘big data’ about the freight traffic in the city, Global Positioning Systems (GPS) devices which are used in vehicles to identify their exact locations, IoT (Internet of Things), blockchain technology, drones, self-driving vehicles, and robots [27–30]. Modern CL is an element of a smart city, whose functioning is based on collecting and processing big data sets, developing smart applications and paying attention to cyber security [31], as well as the increasing use of AI-based solutions [32]. Effective use of data recording tools requires a broader view of the processes taking place in the city and appropriate information management. It is stressed that city authorities should establish an infrastructure fostering an open IT environment that enables third-party entities to retrieve anonymised city data. This includes information collected by smart city sensors e.g. cameras [33]. The development of the smart city concept is not only determined by the application of technological factors, but also by further groups of factors, i.e. human factors and institutional factors [34]. The notion of pillars, or enablers of smart city, is also well established in the literature, among which the following are mentioned: institutional infrastructure, physical infrastructure, social infrastructure, and economic infrastructure [35]. Without these, it is not possible to successfully develop smart CL, but also other components of a smart city e.g. smart healthcare or smart waste management and others individually developed depending on the city.

At the same time, the close connection between technology and sustainable development of the city is emphasized. Extensive use of technology in cities generated the creation of the relatively new concept of a *smart sustainable city* [36]. The term is the result of mutual interconnections between trends resulting from sustainable growth, urbanisation and ICT [37]. One of the definitions states that “a smart sustainable city is a city that meets the needs of its present inhabitants without compromising the ability for other people or future generations to meet their needs, and thus, does not exceed local or planetary environmental limitations, and where this is supported by ICT” [38]. Properly functioning CL is one of the dimensions that can contribute to the implementation of the idea of a smart sustainable city.

In some cities, the plan and specific measures for achieving sustainable urban transport are introduced and implemented as strategic documents of the city authorities, i.e. urban transport plans or SULPs (Sustainable Urban Logistics Plans). Sometimes SULPs are separate documents, and sometimes they are part of a city-wide strategy. The creation of SULP is also the subject of international research projects [39].

The logistics measures implemented in modern cities should translate into positive results of the city’s functioning in the social, economic and environmental aspects [40, 41]. Properly selected and skilfully implemented CL tools contribute to the progress of fulfilment of UN SDG 11 ‐ *Make cities and human settlements inclusive*, *safe*, *resilient and sustainable* [42].

CL is at the same time a research area as well as a set of activities and measures that can contribute to improving the quality of life of urban residents and improving the efficiency of other stakeholders. CL is a part of a living city whose continuous development will drive organizational, business and technical changes in the field of urban freight deliveries.

The question arises whether having a huge amount of data by individual stakeholders is sufficient to ensure the development of CL in the spirit of sustainability. All of the previously mentioned examples of solutions used in CL place great emphasis on technological tools, sometimes creating the impression that they are a guarantee of success of the whole CL project. There is also an approach in the literature that, rather than techno-enthusiasm, emphasises the need to pay more attention and develop tools that will allow better communication between stakeholders, better identification of problems and mutual understanding of all stakeholders’ needs. In fact, smart cities depend not only on a city’s provision of hard infrastructure (physical capital), but also on the availability and quality of knowledge communication and social infrastructure (human and social capital) [43]. Contemporary cities are referred to as information hubs and knowledge repositories [44]. Cities collect very large data sets on many different areas of people’s lives. At the same time, much of the collected data is not used to the extent it could. The increasingly advanced practical solutions of CL must be supported by the development of CL as a scientific discipline. When making decisions in the area of CL, it is necessary to take into account technical, economic and social factors. To fully understand and explain the processes of CL, it is necessary to conduct interdisciplinary research. CL should not be seen only as a set of practical solutions to mitigate the negative effects of the rapid development of urban freight transport. The theoretical foundation of CL as well as analysis of the best practices are often supported by such research areas as operations management, operations research (for example optimization of last mile delivery routes), supply chain management and industrial engineering [45]. This paper demonstrates the need to include KM in this set.

That is why, the second research perspective adopted in this study is the KM achievements. The scientific field of KM and the concept of knowledge have evolved over time. As with CL, the use of emerging technologies is inevitable also in this area. Currently, we are dealing with the fourth phase of knowledge production and dissemination named Knowledge 4.0 [46]. The nomenclature refers to the terminology of Industry 4.0 [47]. The determinant of Knowledge 4.0 is a digitised knowledge society, which is characterised by [46]: digitization of everyday life and value creation; cognitive, social, collaborative and networked systems; artificial intelligence; digital penetration of professions and education. KM are processes and practices aimed at improving the effectiveness and efficiency of the management of organizational knowledge resources [48]. KM guidelines and best practices usually focus on enterprises and there is a lack of research looking for these relationships concerning CL.

**Fig 1:**
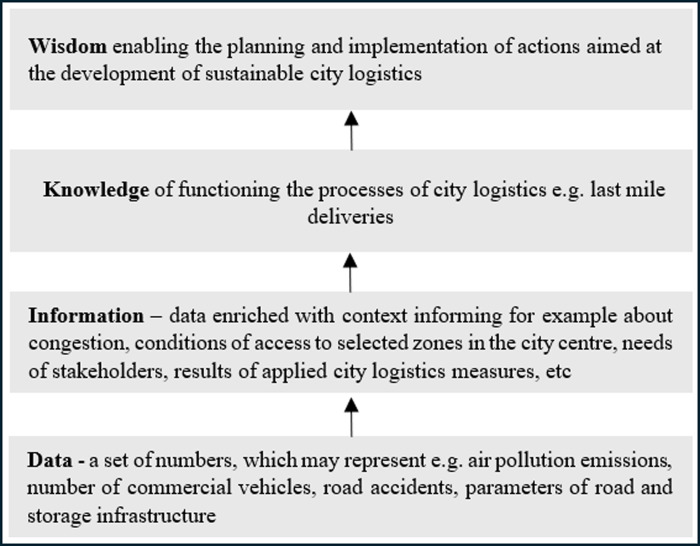
Wisdom hierarchy related to the field of CL. Source: Own elaboration based on WKID concept described among others in [49].

The knowledge hierarchy model, often also called the WKID (Wisdom-Knowledge-Information-Data) pyramid, widely recognized in the scientific work of management and organizational studies, has been presented taking into account the specifics of CL (Fig 1). Data, understood as facts, numbers, words, images or pictures, are then transformed into information, that is, data that has been assigned a context, which in turn are transformed into knowledge [49]. The fourth level–wisdom–is the most abstract. It is explained as understanding and insight needed in the process of decision-making [50]. It is emphasized that not all information becomes knowledge, and increasingly common and easier access to data does not always translate into the wisdom of the organization [51].

Building knowledge resources and moving up the knowledge hierarchy is a long-term and multi-action process. In the literature there are several models that depict KM as a certain cycle that consists of several processes. These elements follow a fixed sequence, which is cyclic as knowledge building is a continuous process. The stages are called differently depending on the approach of the author. Despite these differences, there are also a lot of similarities. For instance, Botha et al. [52] outlined three key processes: knowledge creation and sensing, knowledge organising and capture, knowledge sharing and dissemination. Another model differentiates: knowledge creation process, knowledge transfer, and knowledge embedding processes [53]. In some models, more detailed processes are listed, for example: setting knowledge goals, identifying knowledge, acquiring knowledge, developing knowledge, sharing knowledge, utilising knowledge, retaining knowledge, evaluating knowledge [46]. The differences in the naming of processes mentioned by different authors are largely due to the degree of detail of the analysis and adopted research perspective. Models are created for enterprises and organisations in general terms, without indicating the industry or type of activity.

This study draws on existing knowledge gained from the literature on KM processes in general and enriches it with the specifics of CL activities. Based on the literature and authors’ experience gained in CL projects, four main KM processes have been selected for use in this study: data sources identification (1), data gathering and knowledge acquisition (2), data processing, knowledge creation and application (3), and knowledge sharing and retention (4). The specified process names refer to the levels of the WKID hierarchy and emphasize the need to build knowledge resources on acquired and then processed data. They are examined in detail in the analytic part of the paper.

Building knowledge resources on how CL works requires taking into account all stakeholder groups and the relationships between them. One of the specific features of CL is the multilateral structure and heterogeneity of stakeholders whose interests are partly convergent and partly distinct [54]. In the literature, stakeholders are classified in various ways. For example, Anand [55] identifies four main stakeholder groups: administrators, suppliers, carriers and receivers. In this classification, residents are not listed separately and their interests are represented by the administrators. The literature offers many other, often more detailed, classifications of stakeholders. It is possible to identify up to 20 different groups of them [56, 57].

The benefits that different stakeholder groups derive from the implemented CL measures are also diverse [58]. Stakeholders have different needs and different objectives, so the process of the decision-making will require different information and will be made to meet different requirements. For example, in everyday decisions, a city resident will use information about the location of parcel pick-up points, and the time of arrival of the parcel. The retailer will use information on the availability and prices of transport services, the possibility of using urban consolidation centres, possible congestion or the availability of unloading bays. Moreover, the approach to identifying key stakeholder objectives is slowly changing. A few years ago, there was a clear division between goals aimed at maximizing profits, reported by stakeholders representing business, and goals in the area of improving the quality of life of residents, represented by city authorities and by residents [54]. The need to integrate sustainability concerns into all activities carried out by all stakeholders is now underlined [59]. Social responsibility is not only an addition but the essence and the main premise of the creation of new business models in the area of urban freight transport.

**Fig 2:**
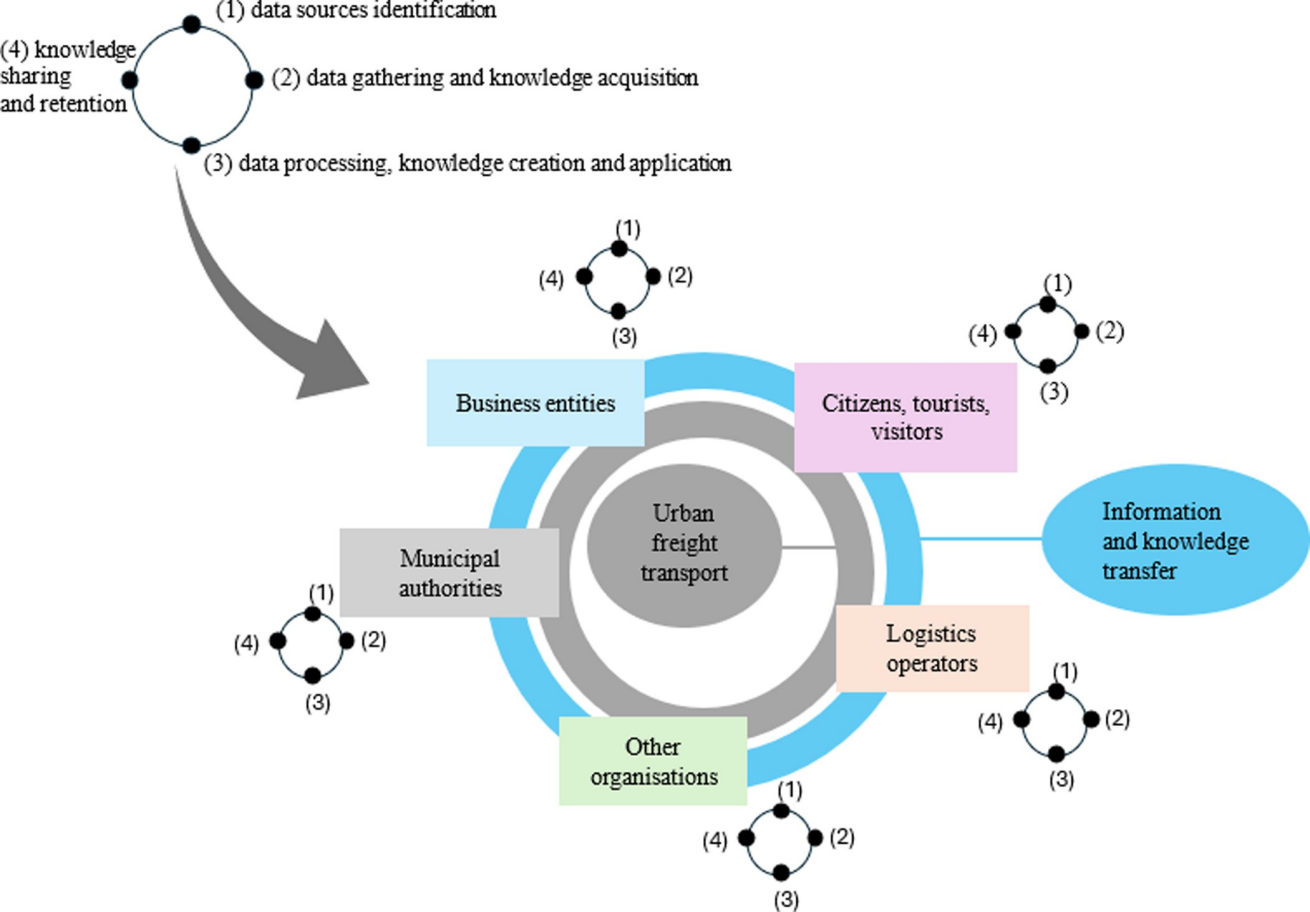
City logistics ecosystem enriched with KM processes. Source: Own elaboration.

CL meets the necessary criteria to be considered an ecosystem [60]. According to an academic approach, the business ecosystem is made up of a total of four basic elements: actors, activities, positions of actors in the system, and links which describe transfers across actors [61]. The CL ecosystem shown in Fig 2 has been enriched by the KM processes, which are implemented at the level of each stakeholder.

Each stakeholder goes through four KM processes building and then using their knowledge resources. Each of the actors implementing logistics processes in the city has its resource of knowledge which are acquired, collected and used for their own use. Each stakeholder has its own KM processes cycle, within which both explicit and tacit knowledge resources are built. At the same time information and knowledge transfer also takes place between stakeholders. In addition, knowledge is often incomplete and is based on many different sources of data and information, the processing of which requires time and commitment. A lack of understanding and knowledge of the needs of other urban actors may hamper the implementation of new CL measures [62].

## Methodology

The paper aims to examine the role of knowledge management in city logistics and identify barriers to the implementation of knowledge-based city logistics. The following research questions are asked during the course of the study:

In which city logistics processes is knowledge management most often used?

Which knowledge management processes in city logistics were most affected by the difficulties?

**Fig 3:**
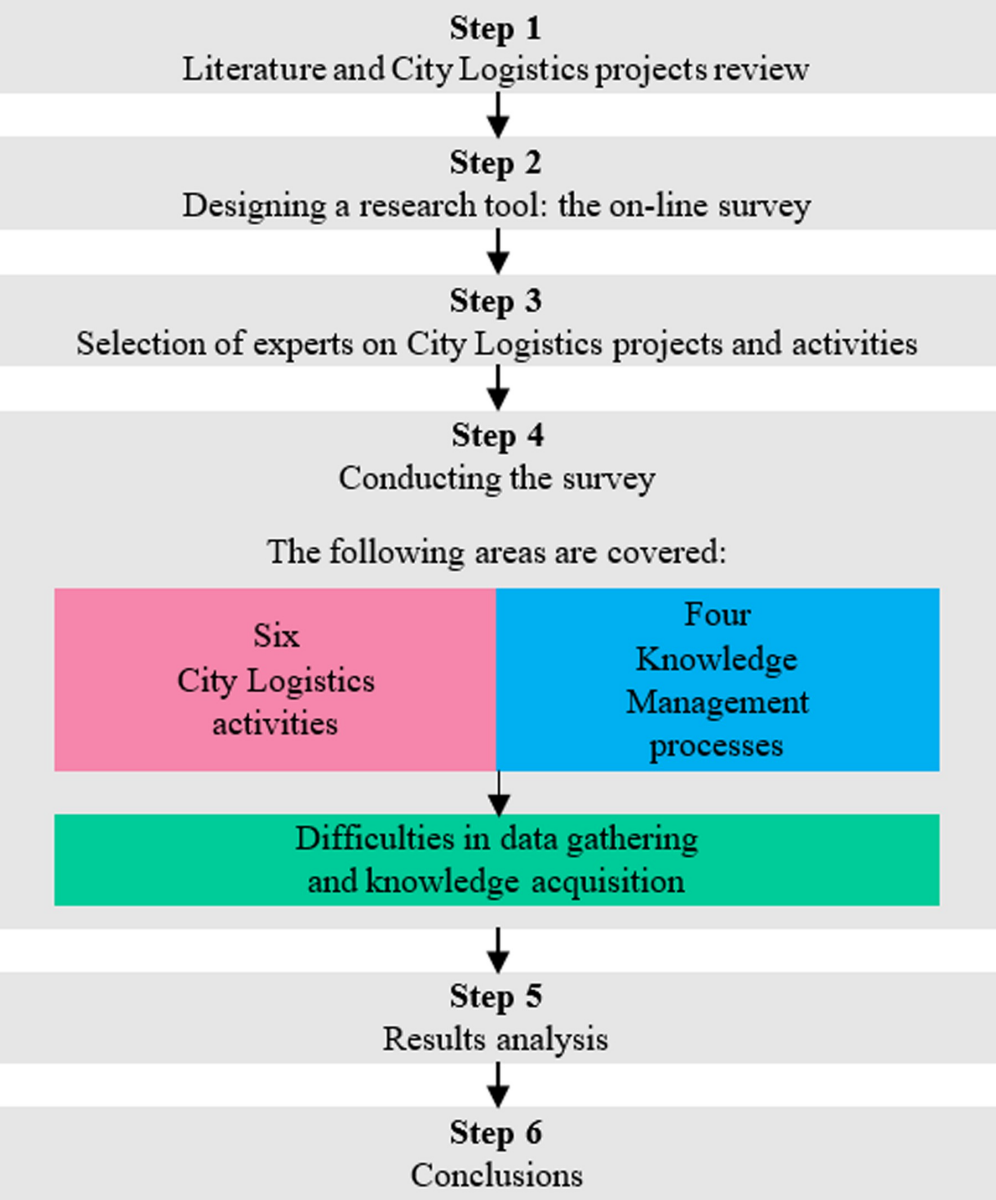
Research framework.

The research were carried out according to the steps shown in Fig 3.

The experts survey was conducted from 1 March to 20 July 2021 using the Computer Assisted Web Interview (CAWI), which is a type questionnaire survey. The questionnaire was developed in Google Forms and mailed to 56 experts selected as a targeted sample. This paper presents only the responses of experts who have experience in CL project work, hence the number of respondents was narrowed down to 31 experts. The number of experts is in line with the methodological recommendations known from the literature [63–65]. Written informed consent was obtained from all experts involved in the study. The characteristics of the experts are presented in Tables 1 and 2.

**Table 1:** Characteristics of experts–years of experience.

Logistics experience in years	Number of experts (N = 31)	Percentage
**Less than 5 years**	4	12,9%
**5–10 years**	9	29,0%
**11–16 years**	7	22,6%
**17–22 years**	4	12,9%
**23–28 years**	2	6,5%
**over 29 years**	5	16,1%

**Table 2:** Characteristics of experts–experience in CL projects.

Projects in which experts were involved
**NOVELOG** ‐ New cooperative business models and guidance for sustainable city logistics;
**CITYLAB** ‐ City Logistics in Living Laboratories;
**EUFAL** ‐ Electric Urban Freight and Logistics;
**LCL**–Low Carbon Logistics;
**C-LIEGE** ‐ Clean Last mile transport and
logistics management for smart and efficient
local Governments in Europe;
**ENCLOSE** ‐ ENergy efficiency in City LOgistics Services for small and mid-sized European Historic Towns;
**U-TURN** ‐ Rethinking Urban Transportation through advanced tools and supply chain collaboration
**SULPITER** ‐ Sustainable Logistics Planning in Central Europe;
**SPROUT** ‐ Sustainable Policy RespOnse to Urban mobility Transition;
**SUMP-PLUS** ‐ Sustainable Urban Mobility Planning: Pathways and Links to Urban Systems;
**ULAADS** ‐ Urban Logistics as an on-Demand Service

Most experts possess over five years of professional experience in CL. Most of them (90.3%) are academics, but they have not only academic experience but have also participated in many international research projects in the field of CL. Experts come from Poland, Norway, Italy, the Netherlands, Belgium, Great Britain, Spain, France, Spain, Croatia, Sweden, Austria, Greece, Ukraine, Turkey and Brazil. Participation in projects was the main criterion for selecting experts. Experts can be considered knowledge workers with a wealth of experience resulting from an in-depth understanding of the specifics of the functioning of many different cities and working in international teams. They gained invaluable knowledge on the specifics and possible difficulties encountered when implementing innovative solutions in different cities. This is an advantage of the selected experts over business experts, who often have more modest experience, sometimes limited to one city.

According to the general guidelines of the KM, one of the ways of acquiring knowledge is the implementation of pilot projects. Selected customers receive a preliminary version of the product for use with a request for its evaluation. The feedback received from them enables modifications to be made to the product. The implementation of CL projects can be compared to this approach. New solutions are being tested in cities with different specificities. Some of the ideas and measures are adopted in a given city on a permanent basis, others do not work and are not continued, or end their lives when the project funding ceases. Regardless of the CL measure lifecycle, the experts who participated in the project accumulate knowledge that enables them to engage in subsequent projects and advise on the selection and implementation of the best solutions, often carried out on behalf of the city authorities. At the same time, it makes them the best possible group of respondents. In conclusion, when selecting experts, we followed the literature which recommend that experts are defined by their understanding of a particular domain, extensive experience and regular engagement with the area of interest [66].

Respondents have experience from being engaged in CL projects in different roles: leader/coordinator, partner/participant and external expert. Nearly half of the participants (45%) took on different roles working on various projects, which gave them the opportunity to assess the complexity of logistics activities from different perspectives.

A CL system can be thought of as a set of activities and projects that are often implemented independently in the city area by various actors. CL is the sum of activities, projects, and business models implemented in a given city. Therefore, the research in this paper understands cities as a fragmented structure and focuses on experts’ opinions on their experience of working in such projects. The object under study is not the entire city, but individual projects and activities that make up the final shape of CL. The object of the research did not cover problems related to project management in general, which recur in projects carried out in many various industries and areas. The research focuses only on the challenges posed by the nature of logistics activities in the city, while the general tasks of project management are outside the research scope of this paper.

**Fig 4:**
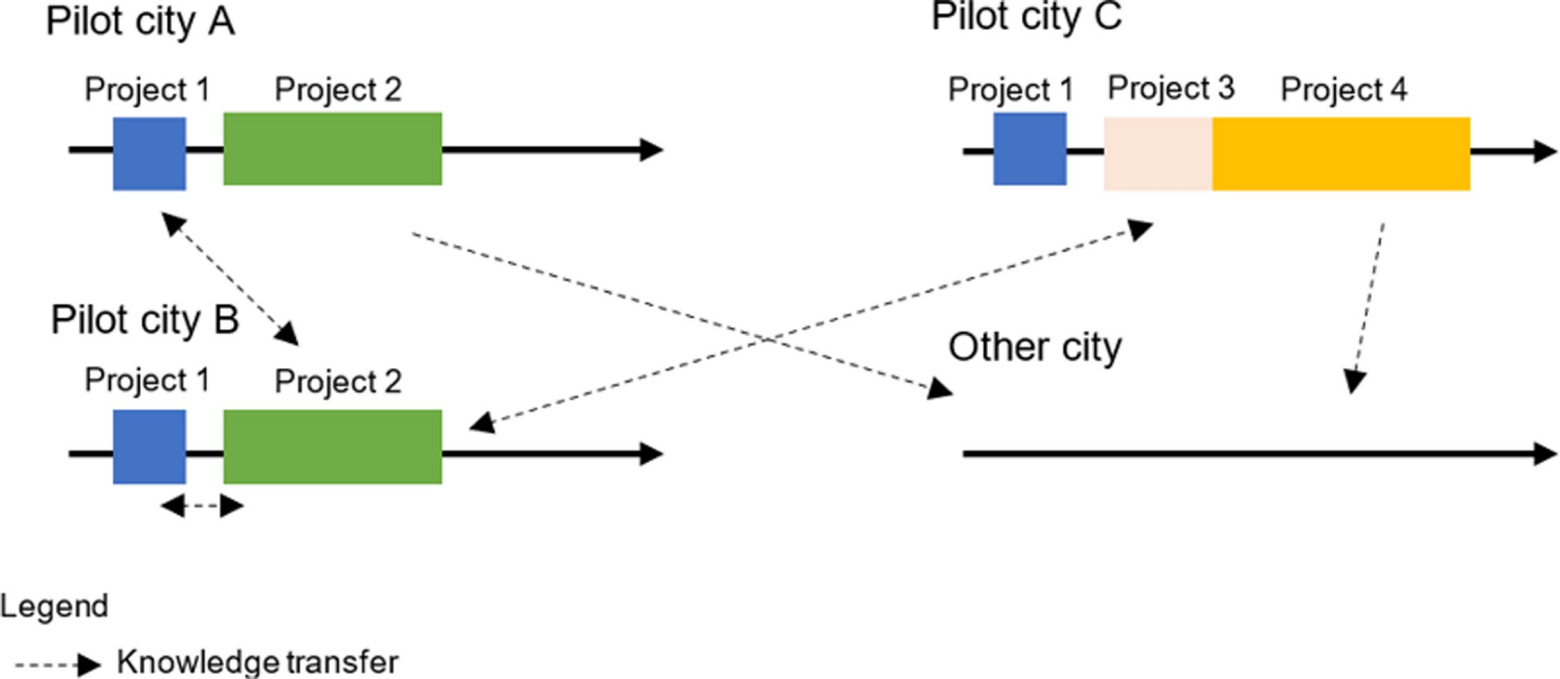
Knowledge transfer in city logistics projects. Source: Own study.

Experts working for research CL projects can be treated as knowledge workers who have the knowledge and experience resulting from the knowledge transfer shown in Fig 4.

Knowledge transfer presented in Fig 4 is possible in several configurations: under a project between several partners and pilot cities (1), between various projects (2), between projects and entities from cities that are not engaged in a given project, as a result of which other cities may avail themselves of the project activities results (3). The Fig 4 shows city C, which is not directly involved in the projects. Nevertheless, it may indirectly benefit from projects carried out in other cities. This is possible thanks to knowledge transfer. The results of the projects in the form of descriptions of best practices, successfully completed case studies or recommendations of experts can be used in other cities, even those not directly involved in CL projects.

## Research results

The research collects and analyses expert opinions on the importance of knowledge management processes in CL. In addition, extensive research material was obtained on the difficulties encountered during the implementation of CL projects tasks and their most common causes.

The respondents were asked several questions. The first question was about the CL processes in which the knowledge approach was applied. The experts analysed six possible processes closely connected with logistics activities and decisions. The 5-point Likert scale was provided for the answers. The respondents assessed the frequency for each CL process and could give the following ratings: 1 –never, 2 –rarely, 3 –sometimes, 4 –almost every time, 5 –every time.

**Fig 5:**
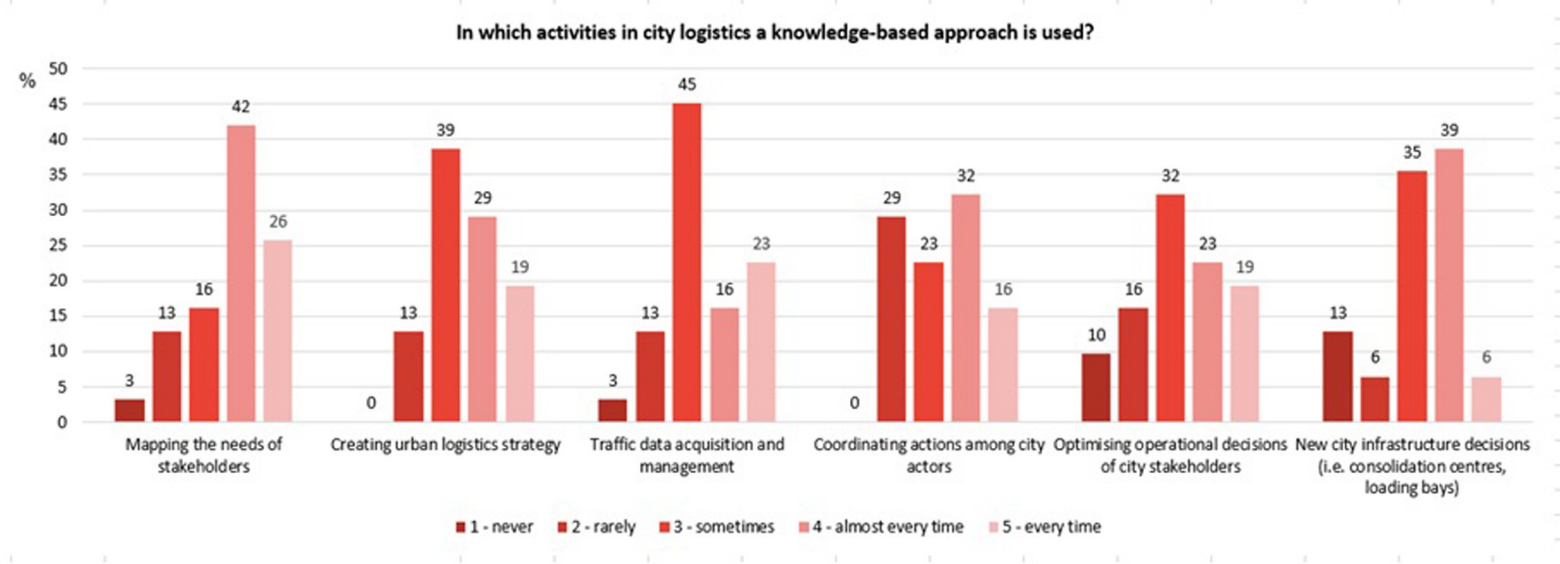
Activities in city logistics in which a knowledge-based approach is used–detailed experts’ responses. Source: Own study.

The results made it possible to assess whether a knowledge-based approach is applied in the indicated areas and to rank them in order to indicate the one which uses such an approach most often. The results of central tendency are summarised in Table 3 and in Fig 5 as a bar chart. The average values are quite high, which shows that in general the knowledge-based approach is important and indeed applied in all areas, although not to the same extent in all of them. The highest average value characterises the area *Mapping the needs of stakeholders*. This is the only sentence with a median of 4 which means that 50% of respondents answered *almost every time* and *every time*. This indicates that respondents are convinced of using a knowledge-based approach in this area. When it comes to *decision-making on new city infrastructure*, experts had the greatest doubts. On the one hand, respondents were most likely to say that knowledge was used almost every time and, on the other hand, a significant 13% of experts felt that the knowledge-based approach was completely absent in that field.

**Table 3:** Activities in city logistics in which a knowledge-based approach is used.

s/n	Areas among the city logistics projects/activities	Average	Standard Deviation	Median	Mode
**1.**	Mapping the needs of stakeholders	3.74	1.08	4	4
**2.**	Creating urban logistics strategy	3.55	0.94	3	3
**3.**	Traffic data acquisition and management	3.42	1.07	3	3
**4.**	Coordinating actions among city actors	3.35	1.06	3	4
**5.**	Optimising operational decisions of city stakeholders	3.26	1.22	3	3
**6.**	New city infrastructure decisions (i.e. consolidation centres, loading bays)	3.19	1.09	3	4

Source: Own study

The experts were also asked if they could name other areas where a knowledge-based approach is often used. In response, the experts identified 13 such areas which can be divided into 4 clusters. They are presented in Table 4. It should be noted that the experts saw the knowledge-based approach in diverse areas. They underlined the significance of new technologies–both concerning telematics systems and new technologies applied in new vehicles. However, it was not the issue of using new technologies that dominated the experts’ statements. The experts showed a broad view on the KM issues, underlining the significant role played by KM in strategic issues such as spatial planning and CL policy, but also pointing to the need to take KM into account in everyday operations. An important area in KM application is also the issue of sustainable city development.

**Table 4:** Additional areas in which knowledge-based approach is often used in city logistics projects/activities.

Group	Areas named by experts
**New technologies**	1. Integration of 5G and ICT technologies (IoT, Blockchain, AI).
	2. New vehicle technology.
**City strategy**	3. Spatial planning.
	4. Strategies for the development of operators of transport services in cities.
	5. Cooperation between stakeholders.
	6. Introduction of city logistics perspective into urban transport and mobility management.
	7. City logistics policy
	8. Development of guidelines / recommendations.
**Sustainability issues**	9. Evaluation of trials; sustainable solutions; policy impact assessment.
	10. Environment and calculating emissions, noise.
**Operational issues**	11. Data on logistics demand and supply.
	12. Freight demand modelling, freight policy.
	13. Rail transport and public transport.

Source: Own study based on expert’s opinions

**Fig 6:**
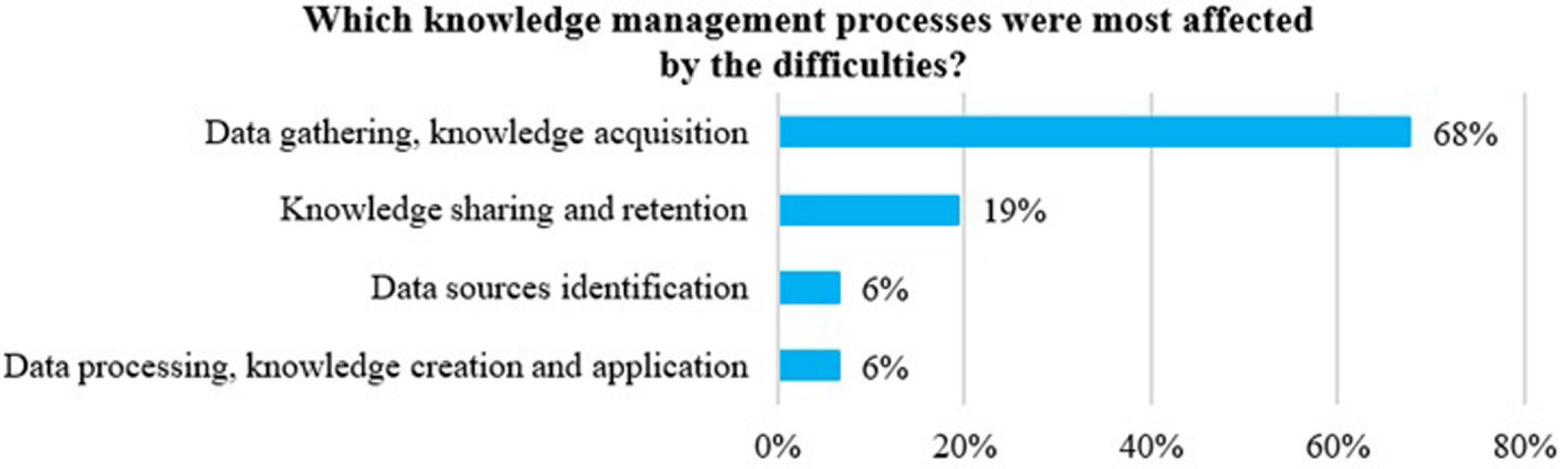
Knowledge management processes in city logistics which were most affected by the difficulties. Source: Own study.

The next two questions relate to the difficulties encountered while working on the project in the context of KM. The experts were asked to indicate which of the identified KM processes were the most affected by difficulties. The results show (Fig 6) that most experts believe that the difficulties mainly concern *the data gathering and knowledge acquisition processes*. This means that with a view to improving KM in a CL project, it is necessary to focus on these processes and look for new organisational solutions for them as well as support in the form of new technologies.

**Fig 7:**
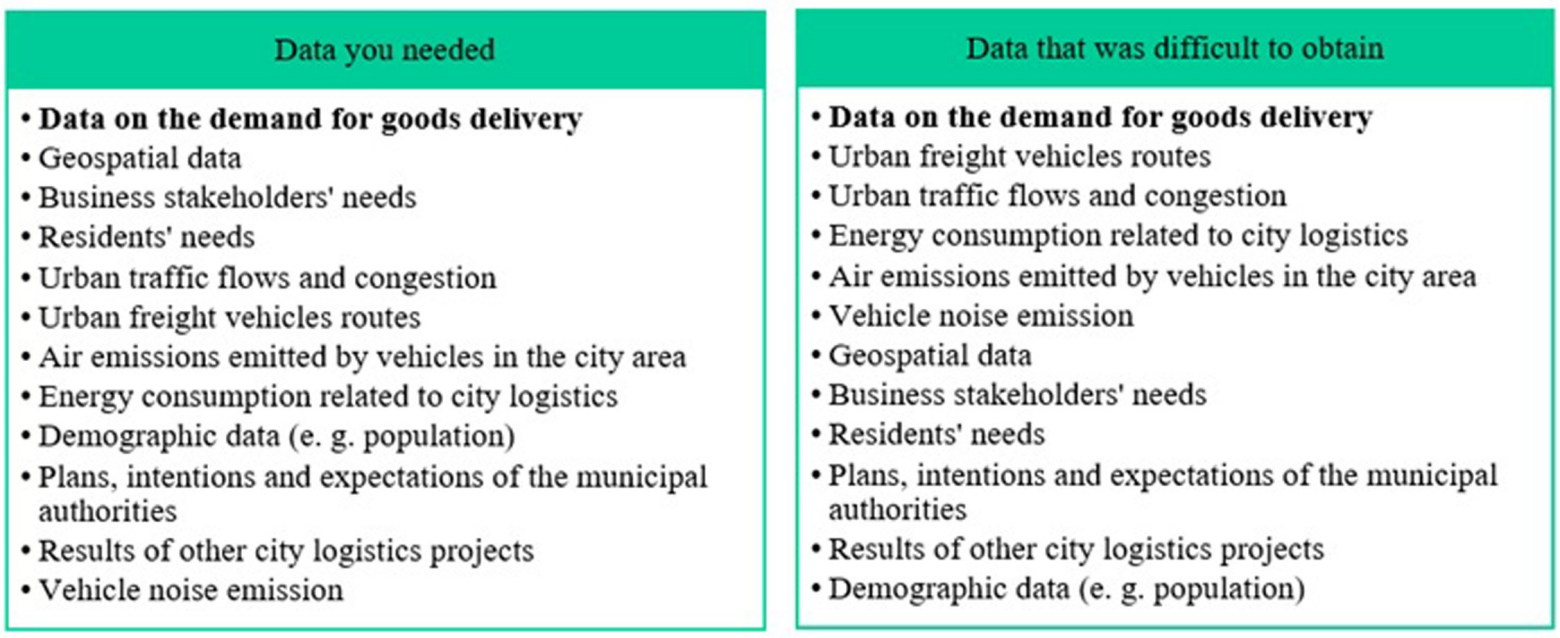
Data needed and data that was difficult to obtain in city logistics in the opinion of respondents. Source: Own study.

The next question posed to the respondents was connected with data gathering and knowledge-sourcing processes, which had been identified as problematic. The respondents were presented with 12 possible categories of data and information on CL. The experts had to decide which data were needed for logistics activities and which data were difficult to access. The results are presented in Fig 7. The data categories are the same in both columns, only the order in which they are presented is different. They are arranged in the descending importance order. The results show the same kind of data in the first position in both columns. Data which are the most needed are at the same time the most difficult to obtain. And that is the data on the demand for goods delivery.

Almost all types of data appeared to be needed to run projects–they all gained more than 50% of respondents’ votes. The least popular category was data on noise emissions from freight vehicles ‐ 48% of respondents voted for this category of data.

Among the hard-to-get data, apart from the data on the demand for goods delivery, data on transport operations was ranked high: vehicle routes, traffic flow, and energy consumption in logistics processes. The data that are the easiest to obtain are those which are not intended exclusively for logistics but are of a general nature–demographic data.

The experts also had the opportunity to write freely about their own experiences concerning difficulties in accessing data. They were asked to identify additional difficult-to-reach data and information. We obtained eleven ideas, which we grouped into three categories. They are presented in Table 5. The most numerous group is related to operational data. The experts noticed that the needed data were not gathered, stored and made generally available. This regards not only the statistical data about freight transport but even the characteristics of road infrastructures in cities. The respondents’ remarks regarding the lack of access to appropriate data will recur in their responses to subsequent questions.

**Table 5:** Difficulties in accessing data and information in city logistics.

Were there other difficult-to-reach data and information?	
**Group**	**Opinions**
**Development, progress**	• Data on various aspects of socio-economic development at the city level are less available in public statistics. This is a big problem both at the level of national statistics and at the level of Eurostat.
	• Effects of projects.
**Data on stakeholders behaviour and motivation**	• Data on stakeholders’ motivations explaining actual and future potential behaviours.
	• Demand side is most difficult and pressing.
**Operational data**	• Delays and operational costs.
	• Customer delivery data (commercial).
	• Specific vehicle fuel consumption data.
	• Economic data regarding the costs of vehicles and their daily business.
	• In some European cities, there was zero data on logistics, as no one had ever done anything or collected any relevant goods transportation data.
	• Combination of commercial vehicle traffic and logistics (you have either traffic, with almost no data on loads, requirements or the other way around).
	• In most cities’ administrations, there is not a single person responsible for logistics. Missing infrastructure and utilisation data extend to such fundamental aspects such as who did what by when, where any commissioned reports and statistics are, or where the camera and loading bays are located, etc.

Source: Own study

The experts underlined the willingness to use the results of previously implemented logistics activities, often carried out based on pilot studies held in other cities concerning similar problems and proposing solutions that are worth testing in various conditions. The survey also checked which areas of knowledge from other projects were used the most often. The experts’ responses are shown in Table 6.

**Table 6:** Experts’ views on usefulness of other city logistics projects.

What knowledge from other city logistics projects do you use?
• I use ideas in the field of research methodology, data sources, and methods of processing.
• Sharing the best practices.
• All … know-how, living labs, methods …
• Definitions, systematics, measurable effects of projects.
• Connection of city logistics with other urban planning issues.
• Their projects and their spatial and flows data.
• Concepts, findings of surveys.
• Strategic recommendations.
• Data needs and results of specific solutions adopted in a given context.
• All knowledge and data from testing new solutions in urban logistics.
• Data on demand.
• Used experiences from the projects listed above.

Source: Own study

The experts’ opinions indicated that they wanted different types of knowledge regarding practically every stage of project life. It is worth noting that the experts did not mention only the results obtained in other projects. Among the wanted information there were also survey results, case studies effects or recommendations as well as information about early stages of project implementation–methods, data sources or concepts.

**Fig 8:**
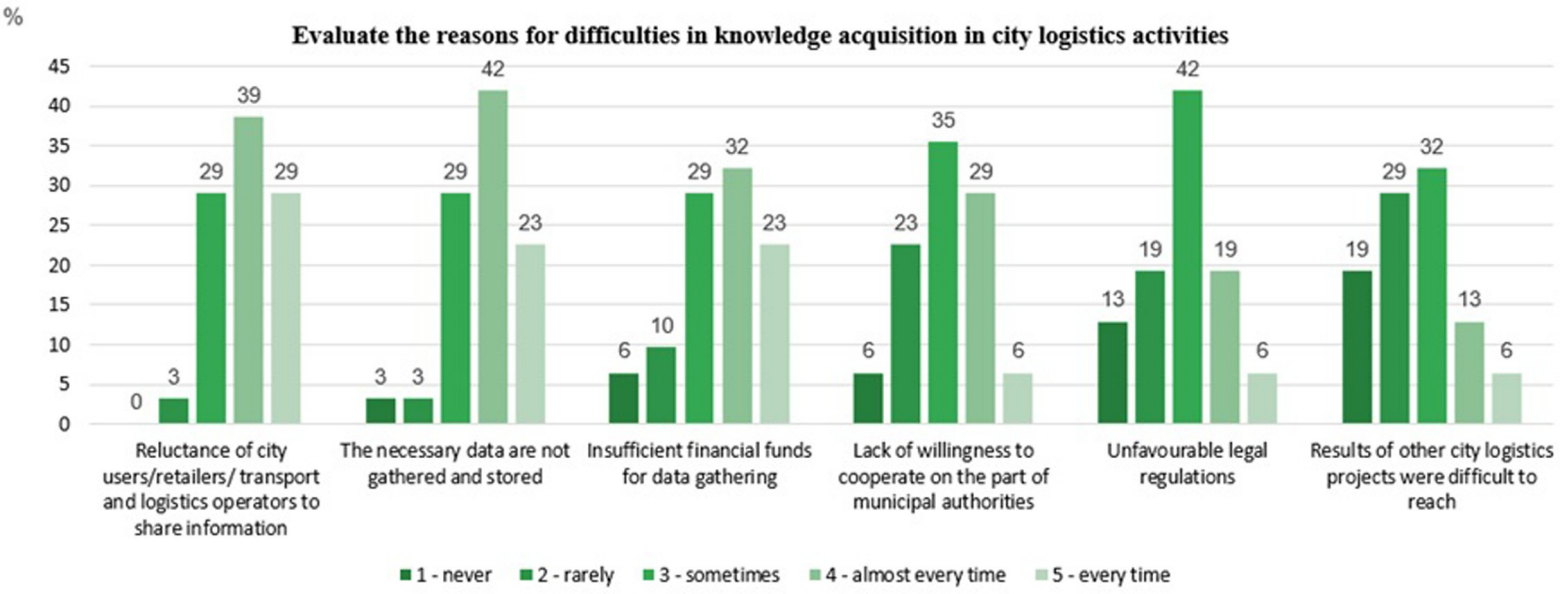
Reasons for difficulties in knowledge acquisition in city logistics activities–detailed results. Source: Own study.

Then the experts were asked to evaluate the reasons for difficulties in knowledge acquisition in CL projects. The experts evaluated the six proposed types of difficulties. The question applied the 5-point Likert scale to assess the frequency of events. The results are presented in Table 7 and Fig 8.

**Table 7:** Reasons for difficulties in knowledge acquisition in city logistics activities.

Reason	Average	SD	Median	Mode
Reluctance of city users/retailers/ transport and logistics operators to share information	3.94	0.84	4	4
The necessary data are not gathered and stored	3.77	0.94	4	4
Insufficient financial funds for data gathering	3.55	1.13	4	4
Lack of willingness to cooperate on the part of municipal authorities	3.06	1.01	3	3
Unfavourable legal regulations	2.87	1.07	3	3
Results of other city logistics projects were difficult to reach	2.58	1.13	3	3

Source: Own study

In the experts’ opinion, *Reluctance of city users/retailers/ transport and logistics operators to share information* was the main reason for the difficulties. It can be seen that there was not a single expert who said NEVER ‐ I don’t see such a problem. The experts were unhesitating when expressing their opinions, which was proved by the low value of standard deviation. The lowest average value was obtained for *Results of other city logistics projects*. 48% of the experts did not experience problems with accessing results of other city logistics projects or experienced them seldom. This is also confirmed by Fig 8, which shows that only 9.8% of experts had problems with access to the results of other projects. However, opinions are divided over this, as 52% of the experts experienced such difficulties sometimes, almost every time and every time.

## Discussion

This study lies at the border of two scientific disciplines–knowledge management and city logistics. Thanks to this, it draws on the achievements of each of them and the results of the conducted research enrich both scientific disciplines. KM as a scientific discipline is constantly actively developed, and research is conducted for new perspectives and contexts [67]. By adopting CL as an object of study, it was possible to enrich management studies and focus on a few important KM issues concerning CL.

Knowledge-based city logistics consists of several activities characteristic of logistics in the city area and at the same time based on processes and rules developed by the achievements of KM. KM processes can be found especially in mapping the needs of stakeholders, creating urban logistics strategy and traffic data acquisition and management. The knowledge-based city logistics include both operational and strategic decision-making.

The results of this study are consistent with the KM literature and confirm that technology is an important implementation factor in the way KM processes are conducted [68], but at the same time tools should only help organizations achieve competitive advantage and should not be used to control the knowledge management pipeline [69].

Logistics solutions aspire to be knowledge-based, which makes CL contribute to strengthening the knowledge economy understood as “production and services based on knowledge-intensive activities” [70]. Of course, the essence of CL is freight transport services, which are the physical flows of goods. Therefore, it cannot be categorised as a knowledge product/service. Literature in the field of KM warns against the hasty typology of services as knowledge services and ill-considered identification of knowledge-intensive industries [71]. Not all service sector employment is knowledge-intensive work. This is the case with a simple transport service. On the other hand, logistics solutions aimed at optimising the movement of goods in the city, greater efficiency of logistics processes, developing and using an alternative power supply in delivery vans, testing autonomous solutions, and at the same time making cities more environmentally and socially sustainable are knowledge-intensive. This means that CL solutions should be based on research, supported by data, backed by consultations with different groups of city stakeholders, and the introduction of innovative solutions should go through all processes of KM. Is CL already like that today? Our research shows that much remains to be done. Knowledge workers see the need to develop CL towards an in-depth application of the knowledge-based approach, but at the same time, they are aware that this is a very big challenge. In business practice, not everything is achieved yet.

**Fig 9:**
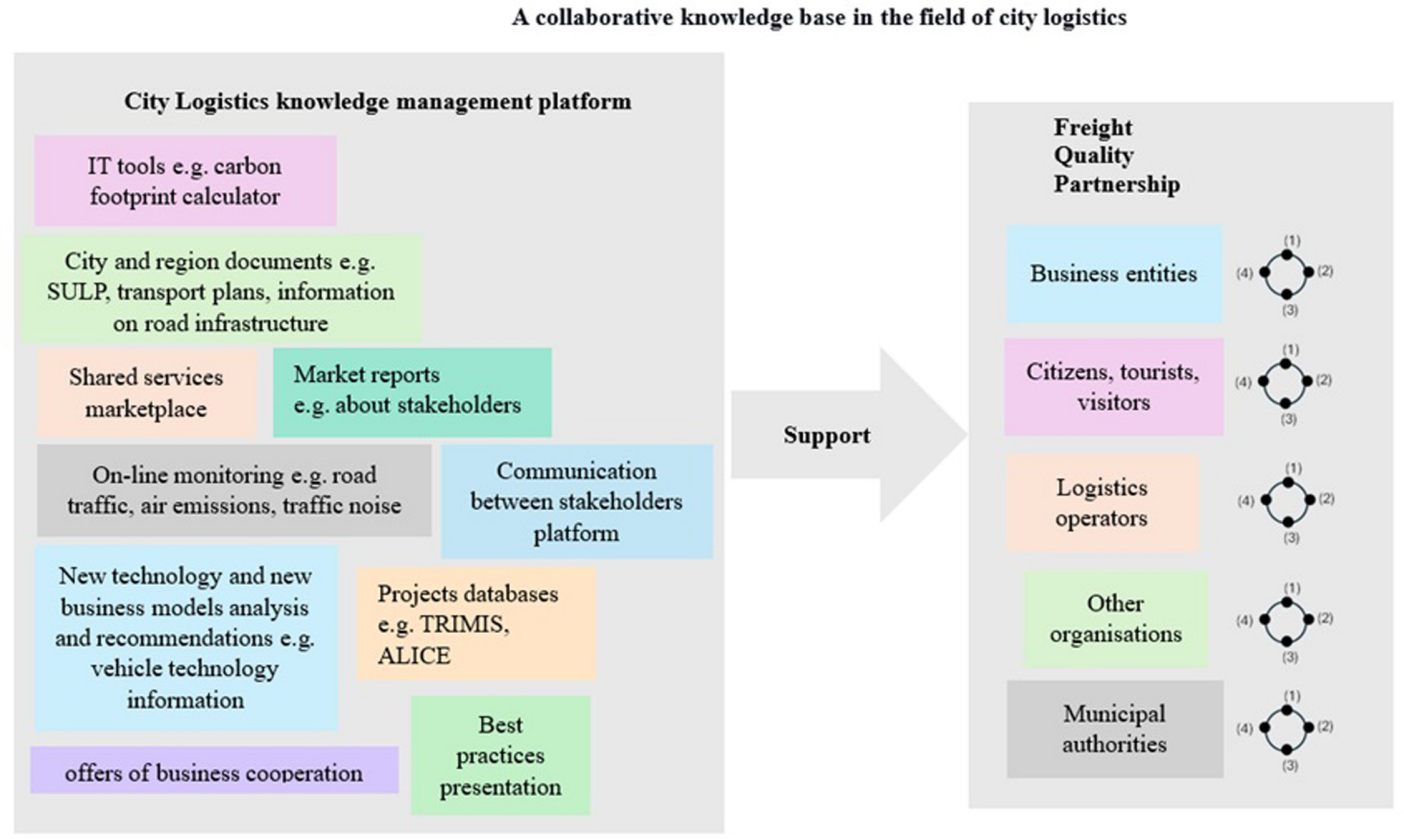
A concept of a collaborative knowledge base in the field of city logistics. Source: Own study.

The implementation of knowledge-based city logistics processes does not always run smoothly. Presented research shows that the biggest problems related to KM processes implemented in CL are the process of data gathering and knowledge acquisition. The solution to the problems identified in the study and at the same time the managerial implication of the conducted research is the proposal to create a CL collaborative knowledge base (Fig 9). The concept is based on two solutions connected with each other. The first is the creation of the city logistics knowledge management (CLKM) platform and the second is the wider use and involvement of CL actors in the Freight Quality Partnership. The synergy between these two solutions is assumed. The expected result of participation in the base is to facilitate the individual inclusion of the obtained insight into the KM processes implemented by each of the CL participants. CL collaborative knowledge base is focused on value co-creation between stakeholders.

The first solution–the CLKM platform–serves as an online repository of knowledge about urban freight transport and topics relevant to CL stakeholders. The main task of the CLKM platform would be to facilitate access to data and information on the broadly understood CL processes. On the one hand, the CLKM platform would act as a typical knowledge repository, where structured knowledge resources in the form of documents, analyses and data sets are stored and made available. At the same time, it would also offer additional functions in the form of links to organisations, e. g. environmental protection organisations, and make available IT tools, e.g. a carbon footprint calculator.

Platforms based on the idea of collaborative knowledge management are a solution known in social, economic and scientific life and are recommended in various areas of business life [72] The benefits of using collaborative Knowledge Management practices are appreciated by companies because they can lead to better integration between supply chain partners and increased knowledge quality, which translates into better organizational performance [73, 74]. Similar benefits can be expected for use in CL.

The results of the survey allowed us to indicate the priority areas that should be included on the platform. The main function modules of the CLKM platform include market reports, best practices presentations, and city and region documents. The CLKM platform would have to be developed on a city-by-city basis, taking into account the needs of stakeholders and the nature of the city. In addition to similar processes carried out in every city, there are also those related to the specifics of, for example, historical cities or port cities. The platform could be an easy way to transfer best practices on CL solutions and share experiences between stakeholders from different cities. The platform would allow benchmarking processes between cities to be easily carried out.

The second solution functioning within CL collaborative knowledge base is FQP. Incorporating FQP into the designed solution, i. e. basing the concept on two pillars at the same time–on the one hand, tools and infrastructure, and on the other, on human resources, is in line with KM theory. According to KM theory, an important part of an organization’s knowledge is not in documents and repositories but in “the minds of knowers” [75]. Regular FQP meetings can help to identify this knowledge and then pass it on to other CL actors.

The organization of FQP is known and recommended in the literature [76, 77]. The regular meetings bring together representatives of the most active stakeholders. FQP aims to discuss and consult public decision-making, as well as to reduce the conflict between different stakeholders’ requirements [78]. Local authorities are usually involved in the organisation and leadership role, often with the support of universities and research units. Thanks to the involvement of the city authorities, it is possible to translate the requirements into regulations and implement appropriately selected CL measures. Experiences of existing FQPs show that they can contribute to increased collaboration between private and public CL stakeholders [79]. As Fig 9 shows, in addition to cooperation within the framework of FQP, each stakeholder implements KM processes themselves in their organizations using the support of the CLKM platform.

## Conclusion

### Theoretical and managerial implications

KM is young and a dynamically developing research field of interdisciplinary character [80, 81]. Its achievements have been developed over the past 40 years [82]. KM is described as a research field which is not dominated by specific topics, methods, and coherent ideas. On the contrary, there is a broad acceptance of a variety of theories and research frameworks [83]. The literature identifies three emerging trends that will determine the future of KM as a research field, which are named extension, specialization and reconceptualization [82]. This study represents the second of these trends.

Specialisation involves decentralisation and creating sub-domains within KM discipline. According to this approach, KM requires the development of specialized studies that take into account the specificities of the research context [84]. Specialization means both the concentration of research on particular issues, such for example the use of data mining in KM, as well as conducting research that are dedicated to specific KM issues typical for example for the public sector, medium-sized companies, start-ups and others. And this is where this study lies. Problems related to the application of KM processes to CL were identified and a solution embedded in CL specificity was proposed.

One of the important issues for KM is the identification of knowledge transfer channels [85]. Our research has shown that R&D projects embedded in the CL area can be such knowledge transfer channels, as the developed solutions and actions undertaken in pilot cities can then be used in other cities. The results are well analysed, described and made public. Our study confirms that knowledge workers responsible for the implementation of such projects often use different types of information and knowledge gained from other projects. They play an important role in the process of knowledge transfer. Adopting the terminology of the theory of diffusion of innovation [86], the experts who participated in our study, and the participants of research projects in the field of CL in general, can be called evangelists or early adopters who contribute to the diffusion of new concepts and solutions in subsequent cities. They help ideas spread between pilot cities and then even further to cities that are not directly involved in the projects, but are interested in learning about new ideas, participating in training and workshops, as well as testing the new solutions themselves.

The aim of the paper was to examine the role of knowledge management in city logistics and identify barriers to the implementation of knowledge-based city logistics. The contribution of the study is to bring KM perspectives to improve CL processes and facilitate knowledge sharing and value co-creation among CL stakeholders. The use of KM is necessary because the ability to use knowledge to make managerial decisions will determine the effectiveness of CL.

The study allowed us to answer the research questions posed. **RQ1** looked for CL processes in which KM is used the most often. The results show that this process is: *mapping the needs of stakeholders*. The next significant ones were: *creating urban logistics strategy* and *traffic data acquisition and management*.

**RQ2** inquired about problems and difficulties that are the most often encountered in the course of implementation of KM in city logistics. It was found that they were mainly concerned with the processes of *data gathering and knowledge acquisition*. Identifying these processes as the most challenging provides valuable insight for managers, municipalities and academics. To enhance knowledge-based city logistics, it’s essential to prioritize these processes and seek out optimal technological solutions as well as new organizational and business support methods. The way to overcome this problem may be convincing the city actors about the multilateral benefits derived from broad access to data. An incentive for this may be the engagement of representatives of city users, retailers, transport and logistics operators in advisory bodies and the conviction that city stakeholders can influence the CL development. The solution to this problem can be a collaborative knowledge base, which consists of two elements ‐ a CL knowledge platform and FQP. A collaborative knowledge base can offer shippers, receivers, logistics operators, and municipal authorities easy access to data, information and knowledge, enabling them to collaborate cooperatively. While IT tools are crucial for gathering data, the focus lies in leveraging this data to establish knowledge resources, thereby enhancing decision-making processes within the city. The proposed solution includes both the benefits of the IT solution and the broader inclusion of the human factor in the process of managing CL.

### Research limitations

Scientific research is always determined by the perspective and research method adopted. This is also the case for this paper. This study does not present all aspects of the knowledge-based city logistics concept, but only a part of it resulting from the adopted aim, the research questions posed, and the research framework adopted. Several further issues need to be addressed within the proposed concept of knowledge-based CL in the next studies. For example, the issue of better motivating stakeholders to share more information about their activities.

Research on the implementation of KM processes was carried out at the level of activities and projects dedicated to CL. No specific city, project or company was analysed, but the broader application of the knowledge-based CL concept was examined. This limitation can also be perceived as an advantage. By examining the opinions of experts involved in many different research projects and pilot studies in many cities, the conclusions are generalized and can be universally applied.

Another limitation of the paper is that it offers only general guidance to stakeholders rather than a specific and detailed plan to be implemented. Developing such a plan could be the next step. The natural choice of the body responsible for the implementation of the knowledge base is the municipal authority. Their role is to adapt the content of the platform to the needs and specificities of the city.

### Future research directions

In future studies, we plan to use other research methods that will allow us to look at the concept of knowledge-based CL from a slightly different perspective, in particular, we plan to apply a multi-case study and analyze selected cities in terms of ways and effects of building knowledge resources about the functioning of urban freight transport. Future research will focus also on assessing the usefulness of selected emerging technological solutions for enriching the knowledge resources about the city and, following this, the development of sustainable knowledge-based city logistics. We will focus on solutions that allow collecting urban freight data independently of road users, e. g. cameras mounted on drones. Thanks to that, one of the barriers identified in the studies is overcome, i.e. reluctance of city users to share data.

## Supporting information

S1 Data (XLSX)
